# Micellar Curcumin Substantially Increases the Antineoplastic Activity of the Alkylphosphocholine Erufosine against TWIST1 Positive Cutaneous T Cell Lymphoma Cell Lines

**DOI:** 10.3390/pharmaceutics14122688

**Published:** 2022-12-01

**Authors:** Antonios G. X. Trochopoulos, Yana Ilieva, Alexander D. Kroumov, Lyudmila L. Dimitrova, Ivanka Pencheva-El Tibi, Stanislav Philipov, Martin R. Berger, Hristo M. Najdenski, Krassimira Yoncheva, Spiro M. Konstantinov, Maya M. Zaharieva

**Affiliations:** 1 Department of Pharmacology, Pharmacotherapy and Toxicology, Faculty of Pharmacy, Medical University of Sofia, 2 Dunav Str., 1000 Sofia, Bulgaria; 2 Department of Infectious Microbiology, The Stephan Angeloff Institute of Microbiology, Bulgarian Academy of Sciences, 26 Acad. G. Bonchev Str., 1113 Sofia, Bulgaria; 3 Department of Applied Microbiology, The Stephan Angeloff Institute of Microbiology, Bulgarian Academy of Sciences, 26 Acad. G. Bonchev Str., 1113 Sofia, Bulgaria; 4 Department of Pharmaceutical Chemistry, Faculty of Pharmacy, Medical University of Sofia, 2 Dunav Str., 1000 Sofia, Bulgaria; 5 Department of Human Anatomy, Histology, General and Clinical Pathology and Forensic Medicine, Faculty of Medicine, University Hospital Lozenetz, Sofia University “St. Kliment Ohridski”, 2 Kozyak Str, 1421 Sofia, Bulgaria; 6 Unit of Toxicology and Chemotherapy, German Cancer Research Center, D-69120 Heidelberg, Germany; 7 Department of Pharmaceutical Technology and Biopharmaceutics, Faculty of Pharmacy, Medical University of Sofia, 2 Dunav Str., 1000 Sofia, Bulgaria

**Keywords:** cutaneous T-cell lymphoma, TWIST1, erufosine, curcumin, synergy, nanotechnology

## Abstract

Cutaneous T-cell lymphoma (CTCL) is a rare form of cancer with local as well as systemic manifestations. Concomitant bacterial infections increase morbidity and mortality rates due to impaired skin barrier and immune deficiency. In the current study, we demonstrated that the in vitro anti-lymphoma potential of erufosine is diminished by TWIST1 expression and micellar curcumin substantially increases its antineoplastic activity. Pharmacokinetic analysis showed that the micellar curcumin (MCRM) used in our study was characterized by low zeta potential, slow release of curcumin, and fast cell membrane penetration. The combination ratio 1:4 [erufosine:MCRM] achieved strong synergism by inhibiting cell proliferation and clonogenicity. The combined antiproliferative effects were calculated using the symbolic mathematical software MAPLE 15. The synergistic combination strongly decreased the expression of TWIST1 and protein kinase B/Akt as proven by western blotting. Significant reductions in NF-κB activation, induction of apoptosis, and altered glutathione levels were demonstrated by corresponding assays. In addition, the synergistic combination enhanced the anti-staphylococcal activity and prevented biofilm formation, as shown by crystal violet staining. Taken together, the above results show that the development of nanotechnological treatment modalities for CTCL, based on rational drug combinations exhibiting parallel antineoplastic and antibacterial effects, may prove efficacious.

## 1. Introduction

Cutaneous T-cell lymphomas (CTCL) represent a heterogeneous group of rare extranodal T-cell lymphoproliferative disorders (non-Hodgkin’s lymphomas, NHLs) which primarily affect the skin by a clonal accumulation of skin-homing CD4+CD45RO+ helper/memory neoplastic T-lymphocytes [1,2]. CTCL progresses by involving the lymph nodes, blood, and visceral organs [3,4], and many patients develop relapsed/refractory disease with a potentially fatal prognosis [5]. Two of the most important subtypes of CTCL are the cutaneous mycosis fungoides (MF) [6] and the leukemic Sézary syndrome (SS) [7]. Both are characterized by a poor quality of life and may lead to seriously shortened overall survival, especially if an extracutaneous involvement is present. An additional important clinical problem associated with major morbidity and mortality rates is the high frequency of concomitant bacterial infections due to impaired barrier function of the skin and progressive immune deficiency [8]. Axelrod et al. found out that 396 of 478 documented microbial infections in CTCL patients were of bacterial origin, e.g., *Staphylococcus aureus*, and were intimately associated with the disease stage [9]. Other published data indicate that staphylococcal enterotoxins may promote the expansion of malignant T-cells [8].

The current therapy of CTCL is challenging, often empiric, and not typically based on specific molecular alterations due to limited insight into the genetic basis of the disease [10]. Recent research based on next-generation sequencing revealed potentially targetable oncogenic mutations in the nuclear factor κ B (NF-κB) and the Janus Kinase and Signal Transducer and Activator of Transcription (JAK-STAT) signaling pathways whose abnormal activation causes apoptosis resistance [11,12,13]. These cancer-promoting somatic mutations affect transcription factors such as TWIST1 (Twist-related protein 1), thereby altering the T-cell effector function and driving lymphomagenesis into proliferation [14,15]. The TWIST1 transcription regulator plays an essential role in cancer metastasis and is activated by a variety of signal transduction pathways, including protein kinase B (PKB/Akt), STAT3, mitogen-activated protein kinase (MAPK), Ras, and Wnt signaling. TWIST1 is thought to promote tumor progression in MF and SS via the p53 axes for cell G1/S cycle arrest with subsequent inhibition of the c-myc-induced apoptosis [16,17], which makes it an attractive molecular target in the personalized treatment approach.

The current therapeutic modalities of CTCL include skin-directed treatments (for patients with limited skin disease and favorable overall survival), retinoids and histone deacetylase inhibitors for advanced-stage disease, and classical cytoreductive chemotherapeutics for relapsed/refractory CTCL forms [7,18,19]. Extracorporeal photopheresis, which is characterized by an excellent side effect profile and moderate efficacy, is considered the first-line therapy for erythrodermic MF and SS [20,21]. Patients with significant nodal, visceral, or blood involvement are generally treated with biologic-response modifiers before escalating to systemic, single-agent chemotherapy. In highly-selected patients, allogeneic stem-cell transplantation may be considered, as this may be curative in some patients [22]. Systemic treatment for relapsed/refractory CTCL has historically relied on traditional chemotherapeutics, retinoids interferons, interleukins, phosphorylase inhibitors, histone deacetylase or proteasome inhibitors; however, responses are often short-lived [23]. Response rates of the clinically approved histone deacetylase inhibitors romidepsin and vorinostat are typically <35%. They can induce some durable responses in heavily pretreated patients and alleviate bothersome symptoms, such as pruritus. Failure to cure advanced SS and MF with large cell transformation peripheral T-cell lymphoma has resulted in a search for novel targeted agents, including antibodies and gene modulators [7], such as anti-CD30 antibody-drug conjugate brentuximab vedotin, anti-CCR4 antibody mogamulizumab, and the fusion protein immunotoxin A-dmDT390-bisFv(UCHT1) [5,18]. Nevertheless, none of these drugs were related to distinguished advances in CTCL therapy.

The drug erufosine (ERF) is a third-generation alkylphosphocholine (APC) with favorable pharmacokinetics and a broad spectrum of in vitro and in vivo antineoplastic activities [24,25,26,27,28], as well as antibacterial activity against pathogenic *Staphylococcus aureus* strains in clinically applicable concentrations [29]. In contrast to other APCs [30], ERF exhibits less pronounced cholinomimetic side effects [31] and can be given intravenously due to a lack of hemolytic properties [32]. The comprehensive mode of action of erufosine includes apoptotic and/or autophagy-mediated cell death in a dose-dependent manner, inhibition of the PKB/Akt-Rb and mTOR axes, and induction of G2/M cell cycle arrest through modulation of the cyclin-dependent kinase inhibitor p27Kip1 [28,33,34,35,36,37,38,39,40,41,42,43]. One of the most important advantages of ERF is the absence of bone marrow toxicity [38,44,45], which makes it an attractive candidate for combined chemotherapies. The progenitor of ERF, miltefosine, was tested in phase I-II studies for topical treatment (6% ointment) of cutaneous lymphomas and led to an overall response rate between 58 and 71% and response duration of 12 months without causing myelotoxicity [46,47,48,49,50]. However, more than 50% of the patients developed strong side effects such as erythema, scaling, skin atrophy, local desquamation, and pruritus [46,47,48], and the clinical trials were discontinued. These results raise the issue and give hope that erufosine could be a more suitable option for CTCL treatment, especially in rational synergistic combinations with anti-inflammatory compounds that could ameliorate possible adverse events without compromising the antineoplastic effect.

Curcumin (CRM) is the major active component of the spice turmeric (*Curcuma longa*, Zingiberaceae) [51] with pleiotropic pharmacological effects which has been used for centuries in the Indian traditional medicine as an anti-inflammatory and antimicrobial remedy [52]. The most important cellular target of curcumin is the pro-inflammatory mediator NF-κB which explains the chemoprotective, antiproliferative, anti-apoptotic, and anti-carcinogenic effects of the compound [53,54]. Curcumin is a potent inhibitor of lymphoblasts’ proliferation in CTCL cell lines through modulation of the JAK/STAT and NF-κB signaling and induction of oxidative stress [55,56]. It is also a suitable candidate for combination therapies because of the low cytotoxicity on normal tissues. The cellular uptake of curcumin is higher in malignant cells than in normal; therefore, it is well tolerated in humans [57]. However, curcumin’s clinical application is limited by the low water solubility and fast metabolism after absorption from the gastrointestinal tract leading to low bioavailability. As a consequence, only small amounts of curcumin are detectable in target tissues which can be overcome through incorporation into polymeric micellar systems suitable for cutaneous application [58,59,60,61].

Having in mind the published scientific data on the therapeutic approaches for CTCL and the gaps in this research area, we set ourselves the goal to evaluate in detail the in vitro pharmacological potential of basically new therapeutic modalities for targeted inhibition of signal transduction pathways involved in the carcinogenesis of CTCL such as TWIST1, PKB/Akt and NF-κB. The focus of the investigations falls on the antineoplastic activity and mode of action of rationally selected synergistic combinations between ERF and micellar curcumin (MCRM) in a panel of CTCL cell lines and the potential of such combinations to inhibit the growth and biofilm formation of pathogenic *Staphylococcus aureus* strains. Our specific aim was to demonstrate that (1) ERF is a suitable drug candidate for the treatment of CTCL, but the transcription factor TWIST 1 reduces the efficacy of ERF in TWIST1 expressing CTCL cell lines, and (2) combining ERF with MCRM will increase the antineoplastic effect of both substances, thus leading to a significant TWIST1 inhibition, deactivation of PKB/Akt and NF-κB, and suppression of *Staphylococcus aureus* biofilm formation.

## 2. Materials and Methods

### 2.1. Drugs and Chemicals

Curcumin (#C1386, Mw = 368.385 g/mol), absolute ethanol (#46139), methanol (#322415), methoxy poly(ethylene glycol)-block-poly(ε-caprolactone) (#900649), glacial acetic acid (#1005706 USP), crystal Violet (#C0775), Tris-HCl (#T5941), Sodium Dodecyl Sulfate (#L3771), glycerol (#G5516), DL-Dithiothreitol (#43815), skimmed milk powder (#70166), Tween 20^®^ (#P1379), 5,5′-Dithiobis(2-nitrobenzoic acid) (#D8130), Hoechst 33342 (#14533) and 3-(4, 5-dimethylthiazolyl-2)-2, 5-diphenyltetrazolium bromide (#M2128, MTT dye) were purchased from Sigma^®^ Life Science (Roedermark, Germany). Working solution of Gentamycin (40 mg/L) was prepared through dilution of commercially available stock (amp. 40 mg/mL, 1 mL, Sopharma^®^, Sofia, Bulgaria) in HPLC purified water. Erufosine (Mw = 503.74 g/mol) was kindly provided by Prof. Hans-Jörg Eibl [62] in the form of 20 mM stock solution in 0.9% NaCl and was stored at 4 °C.

### 2.2. Cell Lines and Cultivation Procedure

All three cell lines originated from the American Type Culture Collection (ATCC): HuT-78 (lymphoblasts, Sezary Syndrome, ATCC^®^TIB-161™), MJ (lymphoblasts, Mycosis fungoides, ATCC^®^CRL-8294™), HH (lymphoblasts, cutaneous T cell lymphoma; ATCC^®^CRL-2105™). Cell cultures were maintained at cell density 5 × 10^4^–8 × 10^5^ viable cells/mL under standard conditions (37 °C, 5% CO_2_, humidified atmosphere, Panasonic CO_2_ incubator, #MCO-18AC-PE, Osaka, Japan) according to the recommendations of ATCC for growth media and subculturing rate. The following buffers and media were used in the cell culturing procedures: (1) RPMI-1640 without Phenol Red (#RPMI-XRXA, Capricorn^®^, Düsseldorf, Germany), supplemented with 4 mM L-Glutamine (#G7513, Sigma^®^ Life Science, Germany), 20% fetal bovine serum (#FBS-HI-12A, Capricorn^®^, Germany), 25 mM HEPES buffer solution (#HEP-B, Capricorn^®^, Germany) and 4.5 g/l D-(+)-glucose (#G8769, Sigma^®^ Life Science, Germany); (2) IMDM (#IMDM-A, Capricorn^®^, Germany), supplemented with 4 mM L-Glutamine and 20% fetal bovine serum and (3) Dulbecco’s Phosphate Buffered Saline (PBS, #D8537, Sigma^®^ Life Science, Germany). For all experiments, cells were plated at cell density 3 × 10^5^ viable cells/mL.

### 2.3. Bacterial Strains and Growth Conditions

The methicillin-resistant *Staphylococcus aureus* strain NBIMCC 8327 (MRSA, Bulgarian National Bank for Industrial Microorganisms and Cell Cultures, Sofia, Bulgaria) was used for the testing of combination effects and biofilm assay. The bacterial cultures were maintained at 37 °C under aerobic conditions using Trypticase Soy Broth (TSB, #LQ508) and Agar (TSA, #M1968) purchased from HiMedia^®^, Mumbai, India. For the biofilm inhibition assay Brain Heart Infusion Broth (BHI, # GM210, HiMedia^®^, India), supplemented with 2% D-Glucose (#G8769, Sigma^®^ Life Science, Germany) was used.

### 2.4. Preparation and Characterization of Curcumin Loaded Micelles

The mPEG-PCL copolymer and CRM were dissolved in dioxane at a ratio 10:1 (*wt*/*wt*). The organic solution was gently stirred for 30 min (700 rpm), and after that purified water was added drop by drop. The resulting micellar dispersion was introduced into a dialysis membrane (MW = 6000–8000), which was further immersed in purified water. The outer aqueous medium was replaced 4 times. The size and zeta potential were determined by photon correlation spectroscopy and electrophoretic laser doppler velocimetry (Zetamaster analyzer, Malvern Instruments, Malvern, UK). Freshly prepared micellar dispersions were measured at 25 °C with a scattering angle of 90°. In vitro release of CRM from the micelles was examined by dialysis. Briefly, the micellar dispersion was poured into a dialysis membrane bag (MW = 6000–8000) that was further placed into 100 mL of phosphate buffer (pH = 7) containing 10% ethanol. Samples were withdrawn from the medium outside the dialysis bag, and the concentration of the released CRM was determined by UV-Vis spectrophotometry at λ = 425 nm.

### 2.5. Cell Viability Test

The cell viability was evaluated according to ISO10993-5, Annex C [63] (MTT dye reduction assay). Briefly, prior treatment cells were seeded in 96-well plates (3 × 10^5^ cells/mL) under sterile conditions (Laminar Air Flow Telstar Bio II Advance, Terrassa, Spain), incubated for 24 h until entering the *log*-phase of the growth curve, and treated with erufosine (0–200 µM in serial twofold dilutions) for 24, 48 and 72 h. The schema for the combinations between erufosine and micellar curcumin followed the recommendations of Chou and Talalay for constant drug ratios [64]. All experiments were performed in triplicate, wherein every sample was repeated 4 times. The formazan intensity was measured on an Absorbance Microplate Reader EL-800 (Bio-Tek Instruments Inc., Winooski, VT, USA) at λ = 550 nm (λ_ref_ = 690 nm) against a blank solution (culture medium, MTT, and solvent).

### 2.6. Mathematical Modeling of Cytotoxic Effects after Single Drug Treatment

The calculation of the median single drug effects (median inhibitory concentrations, IC_50_) was performed as published before [65]. Briefly, we coded a nonlinear regression procedure in MAPLE^®^ software of symbolic mathematics based on weighted least squares statistical criterion as an objective function of the search. A numerical optimization algorithm was used to minimize the sum of weighted squares and to find the estimates of best-fitting parameter values. The median-dose model was applied to obtain the “*IC*_50_” and “*m*”, as presented in Equation (1):

(1) FaFu=DoseDmm,

where *F_a_* represents the affected fraction; *F_u_*—the unaffected fraction (1 − *F_a_*) = *F_u_*; *Dose*—the applied drug concentration; *D_m_*—the median-effect dose (in our case *D_m_* = *IC*_50_), and *m*—a slope of median-effect plot (for *m* = 1 the curve is hyperbolic; for *m* > 1—sigmoidal; for *m* < 1—negative (flat) sigmoidal). In addition, we performed response surface analysis (RSA) methodology in order to reveal the predictive power of the model as a function of the parameters “*IC*_50_” and “*m*”. The range of the value changes in the RSA 3D plot was determined based on the standard deviation of the “*IC*_50_” and “*m*” values obtained during the statistical evaluation of the experimental data with the GraphPad Prism software.

### 2.7. Mathematical Modelling of Drug-Drug Interactions in Cell Lines

A computer program in the platform of the symbolic mathematical software MAPLE^®^ was applied for calculation of the combination effects, and the simulation results were compared with calculations of CompuSyn (Paramus, NJ, USA) [64]. The simulations quantitatively evaluated the effects of action of two applied drugs MCRM and ERF (Dose A:Dose B). The combinations schema followed the recommendations of Chou and Martin [64]. Combination ratios 1:2 and 1:4 [ERF:MCRM] were applied. The mathematical equation of CI (combination index) was written as follows for earlier determined IC_50_ values of drugs A, B, and the combination AB:

CI_(alpha = 0)_ = D_1_/Dx_1_ + D_2_/Dx_2_ (2)

CI_(alpha__= 1)_ = D_1_/Dx_1_ + D_2_/Dx_2_ + alpha × (D_1_/Dx_1_) × (D_2_/Dx_2_), (3)

where D_x1_ = dose of drug 1 only to obtain 50% cell inhibition; D_1_ = dose of drug 1 to obtain 50% cell inhibition in combination with D_2_; D_x2_ = dose of drug 2 only to obtain 50% cell killing; (D) 2 = dose of drug 2 to obtain 50% cell killing in combination with D_1_; and the values of alpha = 0 for mutually exclusive or alpha = 1 for non-mutually exclusive actions of drugs. Only the results for mutually exclusive actions of drugs are presented in this study. The step-by-step, fully automated simulation procedure resulted in isobolograms for HuT-78 and MJ cell lines.

According to the theory included in the manuals of the software program CompuSyn [64], the meanings of CI can be classified in more detail as follows: CI > 1.3 shows antagonism; CI = 1.1 to 1.3 moderate antagonism; CI = 0.9 to 1.1 additive effect; CI = 0.8 to 0.9 slight synergism; CI = 0.6 to 0.8 moderate synergism; CI = 0.4 to 0.6 synergism; CI = 0.2 to 0.4 strong synergism. It should be noted that in Equations (2) and (3) the value of D_x_ (dose of a single drug) can be determined from the following equation:

D_x_= D_m_[F_a_/(1 − F_a_)]^1/m^, (4)

where F_a_—stands for the affected fraction (has inhibition); F_u_—means the unaffected fraction “u”, F_u_ = (1 − Fa) (no inhibition, control); D_x_ is the single dose of the drug; D_m_ means the dose giving the mean effect; hence, Dm = IC_50_ (in our case) 50% inhibition; “m”—is the slope of the dose-effect curve.

### 2.8. Determination of Extracellular Curcumin Levels

The extracellular levels of micellar curcumin were determined in comparison to pure reference substance curcumin with a UV-spectrophotometric method using the following system: UV/VIS Spectrometer HP; Diode array detector; Analytical wavelength 426 nm ± 2 nm and operating software. The analytical calculations are based on Multicomponent analysis calculations. The following method options were used: multicomponent analysis (MCA), Beer’s law calibration curve type, least squares fit (LSQ) algorithm, derivative order 0, polynomial degree 0, 1 smoothing point, 2 nm data interval, 426 nm analytical wavelength and temperature of 25 °C. The test was prepared in the following way: for reference solutions (a): accurately weighed masses curcumin RS were dissolved in ethanol to obtain a solution with the following concentrations: C_1_ = 1.00 × 10^−5^ g/mL, C_2_ = 0.75 × 10^−5^ g/mL, C_3_ = 0.65 × 10^−5^ g/mL, C_4_ = 0.55 × 10^−5^ g/mL, C_5_ = 0.50 × 10^−5^ g/mL, C_6_ = 0.45 × 10^−5^ g/mL, C_7_ = 0.30 × 10^−5^ g/mL and C_8_ = 0.20 × 10^−5^ g/mL; For the bioanalytical assay, test solution (b) was prepared as follows: to each sample aliquot containing curcumin were added 4.5 mL solvent mixture from acetonitrile and methanol (1:1, *v*/*v*), the samples were sonicated at 20 °C for 5 min and centrifuged (<5000 rpm, 5 min) for separating of the precipitates, the supernatant mixtures were filtered additionally and subjected to spectrophotometric determination against *blanks* prepared respectively.

### 2.9. Colony Forming Units (CFU) Assay

The clonogenicity survival assay was performed as published before [40]. Briefly, cells were treated as for MTT assay with selected combinations between ERF and MCRM and the responding single drug concentrations and incubated for 48 h. After that period of time, the cells in all treated groups were counted, and 3000 cells/mL from each group were seeded in semisolid medium (0.8% RPMI-methylcellulose, 40% fetal bovine serum), plated in 12-well plates (600 µL/well) in triplicate and cultured for 10 days. Colonies (≥20 cells in clusters) were counted using an inverted microscope (Boeco BIB-100, Hamburg, Germany).

### 2.10. Determination of Reduced Glutathione (GSH) Content

The spectrophotometric assay of Sedlak and Lindsay based on Ellman’s reagent was used to determine the GSH levels after single and combined treatment [66]. Briefly, HuT-78 and MJ cells were seeded at a density of 0.3 × 10^6^ cells/mL, incubated for 24 h, and treated with 20 µM ERF, 80 µM MCRM or the combination of both. After 48 h incubation, 5 × 10^5^ cells/sample were washed with PBR, centrifuged (6000 rpm, 5 min), and lysed in 100 µL lysis buffer (0.5% triton-100 in 0.2 M EDTA) at 4°C (5 min). The proteins were precipitated with 20 µL 20% (*v*/*w*) trichloroacetic acid. The volume of each sample was adjusted to 200 µL with distilled water, followed by centrifugation at 14 5000 rpm (10 min). Supernatant aliquots were transferred in a 96-well plate (100 µL/well) and mixed with 160 µL Tris buffer (0.4 M, pH 8.9) and 4 µL Ellman’s reagent (3.4 mg/mL in methanol). The resulting yellow product was measured at 405 nm on an Absorbance Microplate Reader EL-800 (Bio-Tek Instruments Inc., USA). The GSH levels were presented as a percentage of the untreated control.

### 2.11. Determination of Cytosolic Mono- and Oligonucleosomes

A photometric enzyme immunoassay (Cell Death ELISA kit, #11 544 675 001, Roche Applied Science, Penzberg, Germany) was used for quantitative in vitro determination of cytoplasmic histone-associated DNA fragments (mono- and oligonucleosomes). Briefly, HuT-78 and MJ cell lines were seeded at density of 0.3 × 10^6^ cells/mL, cultivated for 24 h until entering the exponential growth phase, and exposed to 80 µM MCRM or 20 µM ERF or the combination thereof. After 72 h incubation period, cells were counted, and the immunoassay was performed according to the manufacturer’s manual. The absorbance of the reaction product was measured at λ = 405 nm (490 reference wavelength) against a substrate solution blank.

### 2.12. Caspase-3 Activity Assay

HuT-78 and MJ cells were seeded in 6-well plates at a density of 0.3 × 10^6^ cells/mL, incubated for 24 h, and treated thereafter with 20 µM ERF, 80 µM MCRM, or the combination of both for further 48 h. Cells were counted and 1 × 10^6^ cells/sample were washed three times with PBS and frozen at −80 °C. The measurement of caspase-3 activation was performed with the Caspase-3 DEVD-R110 Fluorometric and Colorimetric Assay Kit (#30008, Biotium, Fremont, CA, USA) following the experimental protocol of the manufacturer. The absorbance was measured at λ = 490 nm on an Absorbance Microplate Reader EL-800 (Bio-Tek Instruments Inc., USA). As positive control, HL-60 cells (5 × 10^4^) were used and incubated in hypertonic buffer (10 mM Tris, pH 7.4, 400 mM NaCl, 5 mM CaCl_2_ and 10 mM MgCl_2_) for 2 h at 37 °C.

### 2.13. Detection of Apoptosis with Hoechst Staining

HuT-78 and MJ cells were seeded and treated for the caspase-3 activity assay. Nuclear fragmentation was imaged by staining the cells with Hoechst 33342 (0.1 mg/mL final concentration) for 30 min according to the protocol of Chazotte [67]. Samples were examined under a Nikon TiU fluorescent microscope (UV filter, 200× magnification), and the images were acquired and processed using EZC1 software.

### 2.14. Western Blot Analysis for Protein Expression

The modulation of TWIST1 and related signal molecules, except for NF-κB, after treatment with erufosine or combinations between erufosine and micellar curcumin was tested by immunoblot analysis as published before [36]. Briefly, all three cell lines were seeded in 6-well plates at a density of 0.3 × 10^6^ cells/mL and treated with 12.5, 25, or 50 µM of erufosine for 24 h or as for the caspase-3 assay. Thereafter, cells were washed in PBS and centrifuged for 5 min at 2000 rpm (Eppendorf^®^ microcentrifuge, Hamburg, Germany). Cell pellets (2 × 10^6^ cells) were lysed in a buffer containing 100 mM Tris-HCl with pH 8.0, 4% SDS, 20% Glycerol, 200 mM DTT, and complete protease inhibitor cocktail (#A7779 Applichem, Darmstadt, Germany). Lysates were boiled 10 min and centrifuged at 13,000 rpm for 10 min at 4 °C. Aliquots of 10 µL were taken from the lysates before adding DTT, diluted five-fold in distilled water and quantified for protein concentration with the Pierce BCA Protein Assay Kit (#23225, ThermoFisher Scientific, Waltham, MA, USA). The total protein lysates (50 mg) were subjected to electrophoresis (8% polyacrylamide SDS gels), and proteins were transferred onto PVDF membranes (#IPFL00005 Sigma–Aldrich). The specific antibody labeling was performed in Tris buffer saline supplemented with 0.1% Tween and 1% skimmed milk. The TWIST1 (sc-81417) and β-Actin antibodies (C-2, sc-8432) were purchased from Santa Cruz Biotechnology^®^, Inc. (Dallas, TX, USA), whereas p-Akt^Ser473^ (#4060) and p-Akt^Thr308^ (#13038) originated from Cell Signaling Technology^®^ (Danvers, MA, USA). The antibodies were diluted according to the manufacturer’s instructions. Immunoblots were developed using an HRP-conjugated anti-mouse m-IgGκ BP-HRP (sc-516102, Santa Cruz Biotechnology) or anti-rabbit IgG (#7074, Cell Signaling Technologies, USA) on a C-DiGit Blot Scanner (Li-Cor Biotechnology, Lincoln, NE, USA). The protein expression was normalized based on β-Actin levels by densitometric analysis of the digitized autographic images using the Quantity One 1-D Analysis software 4.6.6. (Bio-Rad, Hercules, CA, USA).

### 2.15. NF-κB p65 ELISA

HuT-78 and MJ cells were plated and treated for the caspase-3 activity assay. The NF-κB p65 activation after single and combined treatment was evaluated using the NF-κB p65 ELISA (#ADI-EKS-446, Enzo Life Sciences (ELS) AG, Lausen, Switzerland). The protocol was performed following the manufacturer’s instructions.

### 2.16. Biofilm Formation Assay

The biofilm formation assay was performed according to the protocol of Stepanovic et al. [68]. Two-fold serial dilutions of the combinations between ERF and MCRM in concentrations ranging from 1.25/5 to 20/80 µM ERF/MCRM were prepared using BHI broth supplemented with 2% glucose (*w*/*v*). The samples were placed in 96-well polystyrene tissue culture plates at a final volume of 100 µL/well. An equivalent volume of MRSA bacterial inoculum (5 × 10^5^ CFU/mL) was added to each well. Cells were incubated aerobically for 24 h at 37 °C under static conditions. The supernatant was discarded, and planktonic cells were removed three times by washing with PBS (250 µL/well). Cells attached in biofilm were fixed with methanol (200 µL/well, 15 min), air dried, and stained with 0.1% crystal violet (200 µL/well, 5 min). Excess stain was rinsed off with tap water and air dried. The biofilm formation was documented microscopically (40× magnification). Thereafter, the stained biofilm was re-solubilized in 160 μL of 33% acetic acid, and the OD was measured at λ = 550 nm. The biofilm inhibitory concentrations (BIC) were calculated with the GraphPad Prism software and presented in graphs by using a nonlinear regression model (dose-response inhibition, variable slope after normalization, and logarithmic transformation of the applied concentrations). The minimum biofilm inhibition concentration (MBIC_50_) was defined as the concentration of the tested drug that led to 50% inhibition of the biofilm formation.

### 2.17. Statistical Evaluation

The experimental data were analyzed statistically with the GraphPad Prism software (Version 5.00, for Windows, GraphPad Software, La Jolla California, San Diego, CA, USA). Each experiment was performed in triplicate. Minimum of three samples for each concentration, the positive, negative, and untreated controls, were prepared. Data were presented as the mean ± SD. One-way and two-way analysis of variance and the two-independent sample Student’s *t*-tests were applied to compare two or more groups. A value of *p* < 0.05 was considered statistically significant.

## 3. Results

### 3.1. Cytotoxic Effects of Erufosine on CTCL Cell Lines

**Figure 1:**
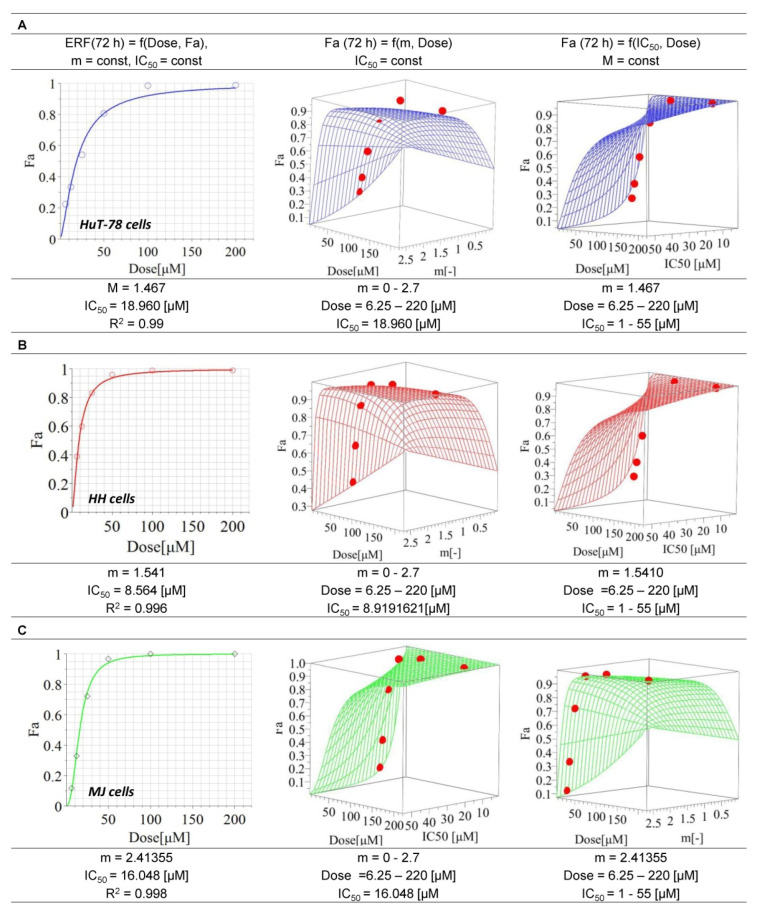
Median inhibitory concentrations of erufosine on the cell lines HuT-78, HH and MJ. **Legend:** ERF—erufosine; m—hillslope; R^2^—coefficient of determination; IC_50_—inhibitory concentration 50% (median inhibitory concentration); Fa—drug effect; (**A**)—Cell line HuT78 (Sézary Syndrome); (**B**)—Cell line HH (cutaneous T-cell lymphoma); (**C**)—Cell line MJ (Mycosis fungoides).

The IC_50_ value of ERF for HH cells (8.6 µM) was nearly twofold lower than that for MJ cells (16 µM) and twice and a half lower than that determined for HuT-78 cells (approx. 19 µM). All coefficients of determination were higher than 0.95, which ensures the best curve fit. The response surface analysis (RSA) confirmed the reliability of the model (Figure 1). No cytotoxic effect (cell viability > 70%) was found for MCRM. 

### 3.2. Production and Internalization Rate of MCRM

**Figure 2:**
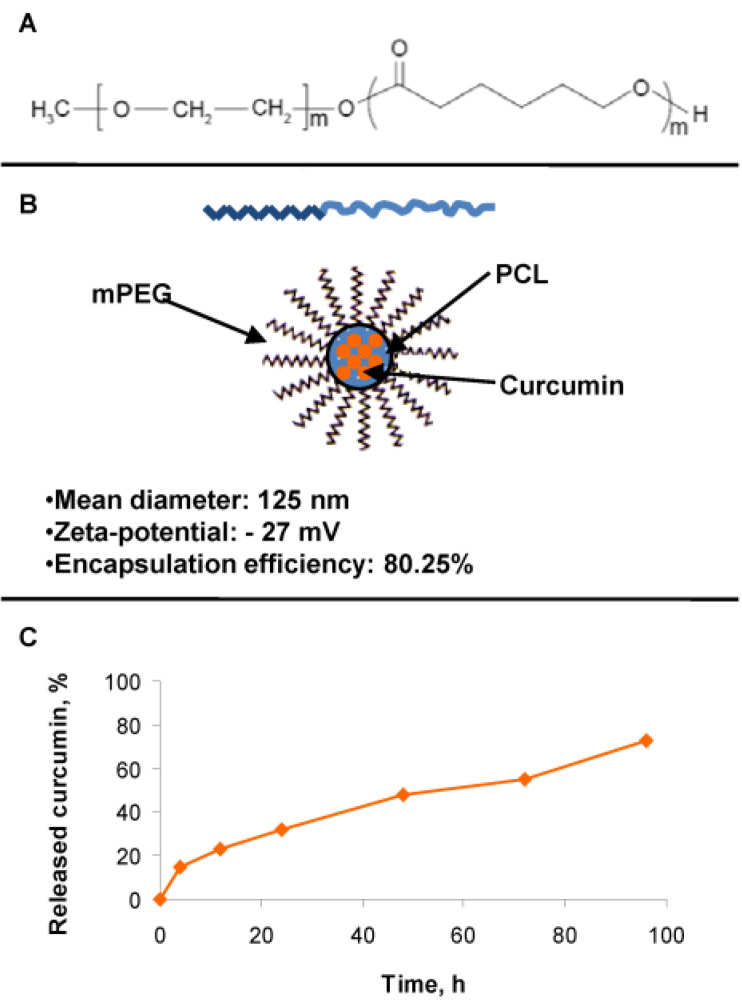
Structure of the micelles and achievement of sustained release with slightly pronounced burst release of curcumin. **Legend**: (**A**)—Copolymer carrier mPEG-PCL; (**B**)—curcumin loaded mPEG-PCL micelles and their physicochemical properties; (**C**)—curcumin release from the loaded mPEG-PCL micelles.

Due to the poor water solubility of curcumin, it was formulated in nano-sized micelles based on methoxy poly(ethylene glycol)-block-poly(ε-caprolactone) (mPEG-PCL) copolymer (Figure 2A). The mean diameter of the micelles loaded with curcumin was approximately 125 nm, and the zeta-potential was negative (−27 mV), providing colloidal stability of the resulting nanosystem. The encapsulation efficiency reached 80%, probably due to a high affinity between curcumin and the hydrophobic block of the selected copolymer (Figure 2B). This fact was related to the achievement of sustained release of curcumin (Figure 2C). In order to quantify the cell internalization of MCRM in comparison to CRM dissolved in ethanol (ECRM), spectrophotometric estimation of the curcumin content was performed in the cultivation medium up to 3 h after the start of treatment (Table 1). Rest concentrations for MCRM were substantially lower than that for ECRM, thus confirming the enhanced internalization of MCRM.

### 3.3. Micellar Curcumin Potentiates the Antiproliferative and Anticlonogenic Effect of Erufosine in TWIST1 Expressing CTCL Cell Lines

**Figure 3:**
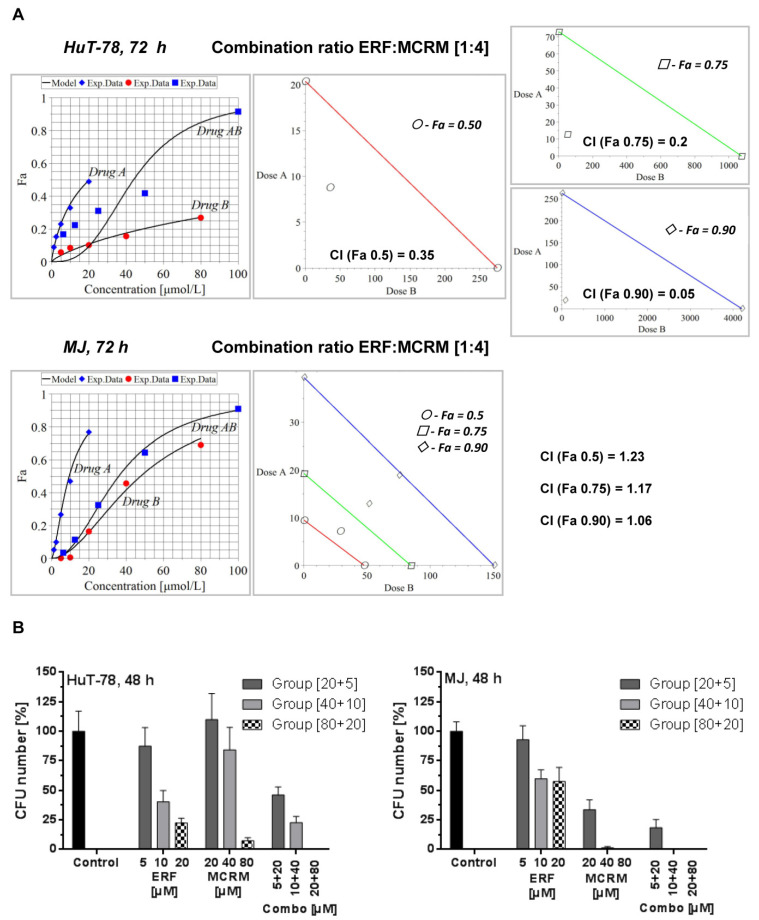
Synergistic and additive combinations between erufosine and micellar curcumin with an inhibitory effect on cell proliferation and clonogenicity of the cell lines HuT-78 and MJ. **Legend:** (**A**)–isobolograms for HuT-78 and MJ cells, calculated by MAPLE software from a MTT assay, where CI stands for Combination Index, Fa 0.5 is the median cytotoxic effect (denoted with points), Fa 0.75 is the effect killing 75% of the cells (denoted with squares) and Fa 0.9 is equal to 90% dead cells (denoted with rhombuses); (**B**)—clonogenic survival (CFU assay) of lymphoblasts from the TWIST1 expressing cell lines HuT-78 and MJ after treatment with combinations achieving Fa 0.9, including the single drug effects of the respective concentrations. Drug A stands for ERF, Drug B stands for MCRM and Drug AB stands for combination between ERF and MCRM.

Based on the calculated median inhibitory concentrations of ERF, we planned the combination treatment following the recommendations and the schema for constant combination ratio in the manual of Chou and Talalay [Manual of CompuSyn. Inc., Paramus, NJ, USA]. The following two combination ratios were investigated: 1:2 and 1:4 [ERF:MCRM]. The highest concentration of ERF used was near the IC_50_ value, and only the concentrations of MCRM were increased up to 80 µM as far as curcumin is less toxic than ERF to normal cells. Only the combination ratio of 1:4 led to synergistic (HuT-78) or additive (MJ) interactions. The results are presented in Figure 3A. The CI values of the combination 80 µM MCRM and 20 µM ERF for HuT-78 cells were lower than 0.9 for all three Fa observed (50, 75, and 90% inhibition of the cell viability), which is indicative of synergism (CI_Fa(0.5)_ = 0.35, CI_Fa(0.75)_ = 0.2, CI_Fa(0.9)_ = 0.17). For MJ cells, an additive effect was achieved by Fa0.9. The data from the CFU test (Figure 3B) revealed 100% CFU inhibition in HuT-78 cells for the combination [20 µM ERF/80 µM MCRM]. In MJ cells, treatment with 40 and 80 µM MCRM and the respective combinations with ERF led to complete CFU inhibition.

The combination indexes for the combination ratio 1:2 from the MTT assay are as follows: (1) HuT-78—CI_Fa(0.5)_ = 1.34, CI_Fa(0.75)_ = 2.28, CI_Fa(0.9)_ = 3.87; (2) MJ—CI_Fa(0.5)_ = 1.57, CI_Fa(0.75)_ = 1.25, CI_Fa(0.9)_ = 1.01. An additive effect was achieved in MJ cells at Fa(0.9). All other variants led to an antagonistic effect.

### 3.4. MCRM Increases the Inhibitory Effect of ERF on TWIST1, PKB/Akt and NF-κB in TWIST1 Expressing CTCL Cell Lines

**Figure 4:**
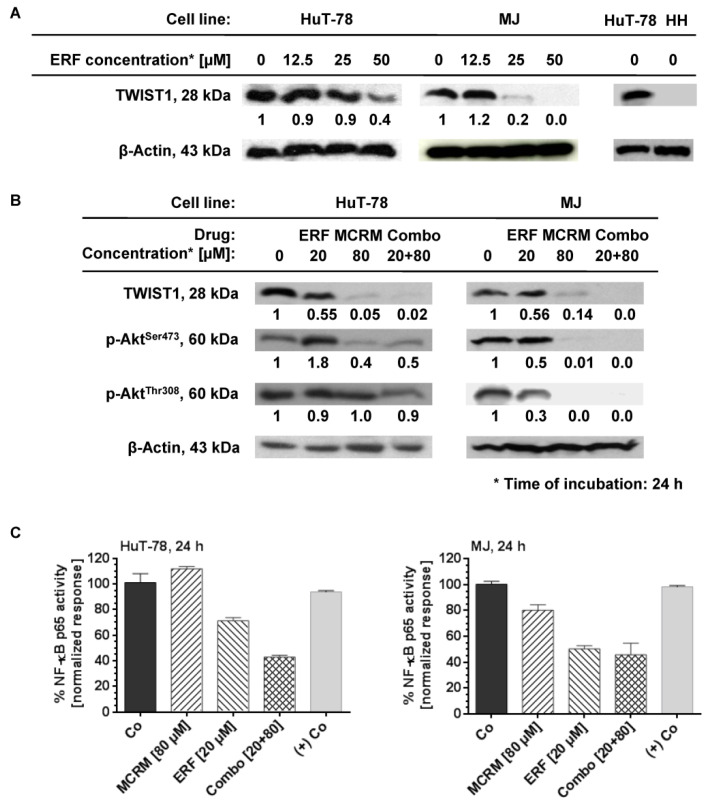
Micellar curcumin potentiates the inhibition effect of erufosine on NF-κB and TWIST1 in the CTCL cell lines HuT-78 and MJ. **Legend:** MCRM—micellar curcumin; ERF—erufosine; (+) Co—nuclear extract of TNFα-activated HeLa cells; Co—untreated control; Combo—combination between erufosine and micellar curcumin with the same concentrations as by the single treatment; (**A**)—expression of TWIST1 by western blot after exposure of the cell lines to erufosine; (**B**)—expression ot TWIST1, p-Akt^Ser472^ and p-Akt^Thr308^ by western blot after exposure of the cell lines to the combinations; (**C**)—NF-κB p65 activity assay of ERF, MCRM and the combinations combination.

No expression of TWIST1 was detected in the cell line HH. HuT-78 and MJ cell lines express TWIST1 and ERF inhibited it in a dose-dependent manner (Figure 4A). ERF alone suppressed the expression of TWIST1 by 60% only at a concentration of 50 µM. In the presence of curcumin, a full TWIST1 inhibition was achieved by using more than a twofold lower concentration of ERF. The same effect was observed in the MJ cell line for single administration of ERF and the additive combination. A single administration of ERF (20 µM) on the MJ cell line inhibited both active forms of the protein kinase B. In the HuT-78 cell line, the dephosphorylation of PKB/Akt at Ser^473^ was not significant against the untreated control, while its dephosphorylation at Thr^308^ was more strongly expressed than the untreated control after treatment with 20 µM erufosine (value close to IC_50_). The combination achieved complete inhibition of protein phosphorylation at both amino acid residues in the MJ cell line, whereas, for HuT-78, this effect was poorly expressed and was the same as after a single treatment with ERF. The NF-κB p65 activity was significantly inhibited (up to 60%) in both cell lines 24 h after administrating the combination.

### 3.5. MCRM Potentiates the Anti-Apoptotic Effect of ERF in TWIST1 Expressing CTCL Cell Lines

**Figure 5:**
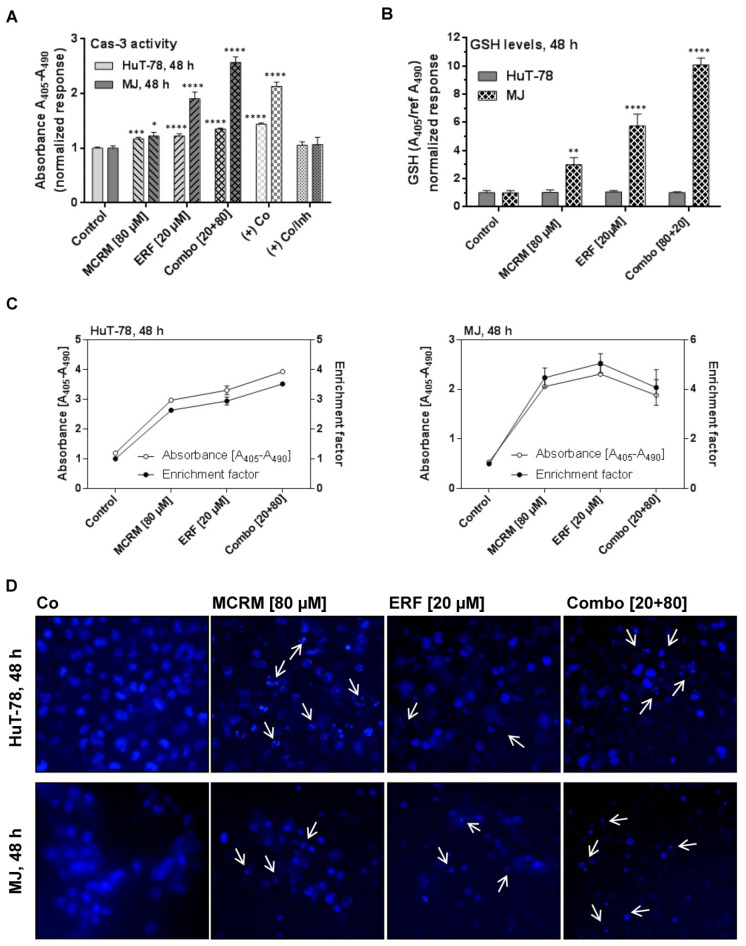
Synergistic interactions between micellar curcumin and erufosine in ratio 4:1 lead to apoptotic cell dead in HuT-78 and MJ cells. **Legend**: (+) Co—cells incubated with hypertonic buffer; (+) Co/Inh—cells incubated with hypertonic buffer in the presence of caspase 3 inhibitor; (**A**)—caspase 3 activity of samples treated with ERF, MCRM or combinations; (**B**)—GSH levels in the same samples, presented in part A; (**C**)—accumulation of mono- and oligonucleotides in the cellular cytosol in the same samples presented in parts A and B; (**D**)—nuclear fragmentation after Hoechst staining of the cells in the same samples presented in parts A, B and C (depicted with white arrows). Significant differences between groups in (**A**,**B**) are marked with asterisks: *—*p* ≤ 0.05; **—*p* ≤ 0.01; ***—*p* ≤ 0.001, ****—*p* ≤ 0.0001.

In both cell lines, the combinations between MCRM and ERF induced apoptosis and resulted in significant nuclear fragmentation in the treated cells to a greater degree than after single administration of the corresponding concentrations. The caspase-3 activity induced in MJ cells by the combinations was twice as strong as compared to that induced by MCRM or ERF separately (Figure 5A). The effect of the combination in HuT-78 resulted in a fourfold higher accumulation of mono- and oligonucleotides in the cytosol of the treated cells as compared to the untreated control (Figure 5C). The Hoechst microscope images (Figure 5D) revealed nuclear fragmentation at preserved nuclear membrane in HuT-78. In MJ cells, a breakdown of cellular structures and extracellular condensed nuclear fragments were observed.

### 3.6. Effects of the Combinations between ERF and MCRM on Total GSH Levels in TWIST1 Expressing CTCL Cell Lines

Glutathione levels did not change at the 48th hour of the treatment in any of the HuT-78 samples, unlike the other cell line MJ, which showed a strong enhancement of total GSH after treatment with MCRM and the combination (Figure 5B). The result correlates with increased levels of caspase-3 in the same samples. Obviously, in MJ cells, glutathione levels were significantly enhanced—up to 10-fold higher than in the untreated control. A concentration-dependent manner of the effect was detected.

### 3.7. The Combination Ratio of 4:1 between MCRM and ERF Inhibits Biofilm Formation of MRSA

**Figure 6:**
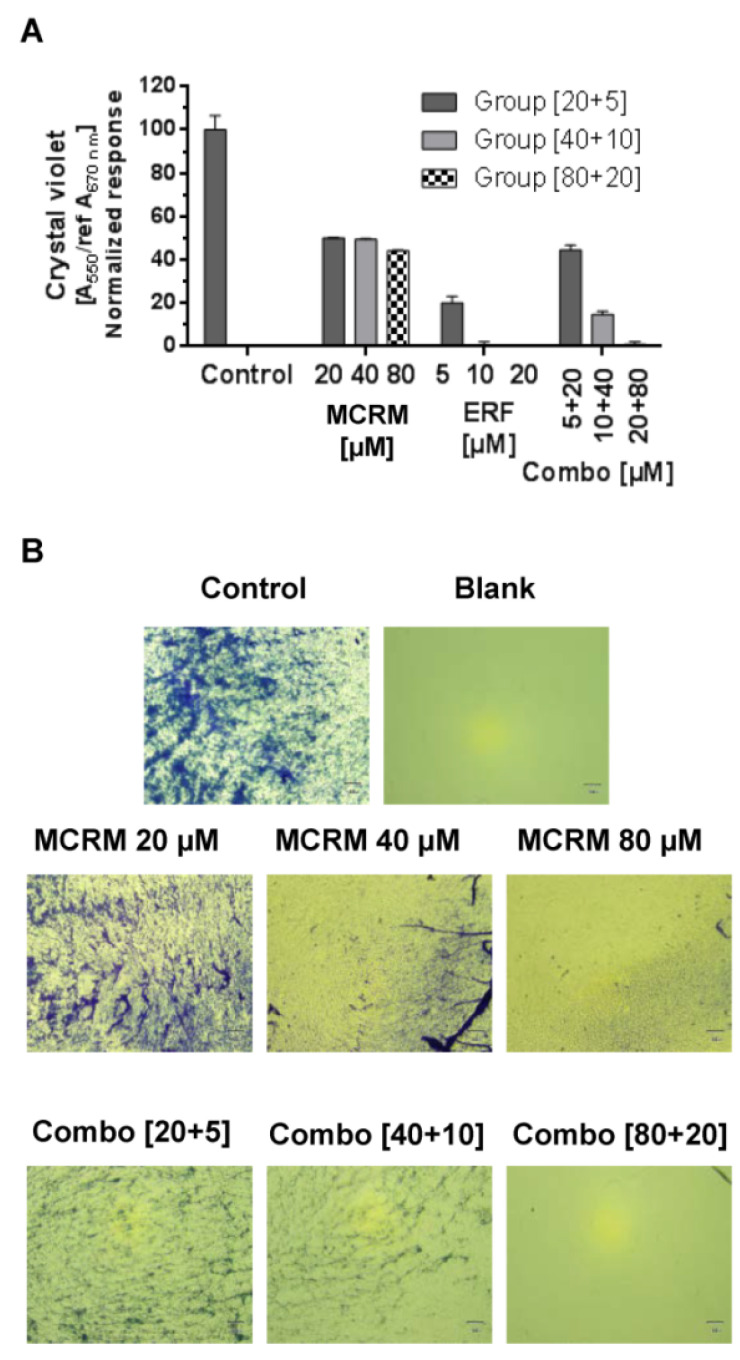
Effect of the synergistic combination [ratio 4:1] between micellar curcumin and erufosine on the biofilm formation of MRSA. **Legend:** ERF—erufosine; MCRM—micellar curcumin; (**A**)—crystal violet absorbance of MRSA biofilm; (**B**)—Morphological evaluation of MRSA biofilm (40× microscope magnification).

The synergistic concentration MCRM:ERF [4:1] significantly inhibited the biofilm formation of MRSA. The effect of the combination of concentrations 80 µM MCRM and 20 µM ERF is equal to that of ERF after a single application, demonstrating full biofilm eradication (Figure 6). In lower concentrations of both drugs, the effect on biofilm formation was antagonistic.

## 4. Discussion

In the present study, we compared and evaluated in detail for the first time, to our knowledge, the in vitro efficacy of the alkylphosphocholine ERF in combination with curcumin incorporated in micellar formulation with a prolonged release on T-cell lymphoma cell lines with and without expression of the oncogene TWIST1. The transcription factor TWIST1 is not detected in normal peripheral blood mononuclear cells. Any increase in its expression levels is abnormal and has been identified as a marker of metastasis and poor prognosis in patients with MF and SS [69]. Furthermore, it has been found that its expression increases in advanced MF/SS lesions and triggers chemotherapy resistance [16,70]. The underlying mechanisms are related to the inhibition of both p53 and Rb tumor-suppressor pathways and the up-regulation of protein kinase B (PKB/Akt) in malignant cells with increased TWIST1 levels [70,71]. In cancer cells, TWIST1 targets several enzymes from the DNA damage response pathway, thus neutralizing senescence and cell death [72]. Based on the accumulated knowledge about the mode of action of the APCs [40,73] and our previous studies on the effects of ERF on CTCL cell lines demonstrating clear inhibition of PKB/Akt [41], increase in the Rb expression levels in T-cells, modulation of the Rb-protein signaling pathway [40] and induction of apoptosis, we presumed that the expression levels of TWIST1 might affect the activity of ERF or vice versa. In our study, ERF showed different activity in the tested cell lines, with HuT-78 being the most resistant and HH being the most sensitive (Figure 1), as evidenced by the estimated IC50 values. The behavior of the RSA simulations, along with the dose-effect curves, confirmed the reliability of the chosen mathematical model in the MAPLE software. The deviation of the model from the experimental data (see points) for the selected 95% confidence interval was not very high, meaning that the values of all constants in the selected ranges of m and the IC50 parameters can be considered reliable and stable. Determination of the TWIST1 expression levels in all three cell lines through western blot analysis revealed that the protein was expressed in HuT-78 and MJ but not in the most sensitive cell line HH (Figure 4). Moreover, inhibition of TWIST1 occurred in MJ at a lower ERF concentration (25 µM) than in HuT-78 (50 µM). This finding could explain the differences in the sensitivity of the cell lines toward the drug.

According to published data, there is cross-talk between TWIST1 and PKB/Akt in the promotion of metastasis via the TGF-β signaling axes [71]. On the one hand, TWIST1 mediates the phosphorylation of PKB/Akt, thus contributing to uncontrolled cell proliferation and invasion. On the other hand, TWIST1 associates directly with the subunit RELA of the pro-inflammatory factor NF-κB to activate the transcriptional activity of the latter and to promote cell invasion through IL8 production [74]. ERF is a potent inhibitor of the PKB/Akt signaling pathway in leukemic and lymphoma cell lines [35,36,37,39,41], whereas the natural product curcumin is a well-known proteasome inhibitor of NF-κB [75,76,77,78]. Based on these facts, we built our hypothesis on the presumption that a combination between ERF and MCRM will achieve simultaneous down-regulation of PKB/Akt and NF-κB with subsequent inhibition of cell proliferation and induction of apoptosis. In addition, ERF and CRM possess in vitro antimicrobial activity against the pathogenic bacterial species *Staphylococcus aureus* [29], which was shown to accelerate the pathogenesis of CTCL [79,80]. That is why; we set the additional goal to study the MRSA anti-biofilm activity of the selected combinations, as far as it is a highly important mechanism providing survival of the microorganisms in conditions of external stress and chemotherapy. Taking into account both the poor curcumin solubility and its hydrolytic instability in a slightly alkaline medium, we assumed that polymeric micelles would be appropriate nano-carriers for cutaneous application. The hydrophobic nature of their micellar core enables the loading of active substances with poor water solubility. Moreover, the loading of unstable drugs into micelles would increase their stability against different degradation processes such as hydrolysis or oxidation [58,59,60,61]. Despite the advantages of the micelles, the studies of polymeric micelles intended for the cutaneous application of curcumin are limited to a few reports [81,82]. Therefore, in the present study, we developed curcumin-loaded nano-sized micelles based on the methoxy poly (ethylene glycol)-block-poly (ε-caprolactone) (mPEG-PCL) copolymer carrier, selected for its biocompatibility, biodegradation and suitable properties for loading of hydrophobic drugs (Figure 2). The nanoparticles were internalized into the treated cells significantly faster than pure CRM (Table 1), which is a prerequisite for the successful cytopenetration and the subsequent intracellular release of curcumin.

The tested interactions between ERF and MCRM in combination ratio 1:4 [ERF:MCRM] were proven to be synergistic for HuT-78 cells and additive for MJ cells (Figure 3A) in difference to the combination ratio 1:2, which led only to additive effects. As a final result, all CI values regarding the cell line HuT-78 were lower than 0.4 for the combination ratio 1:4, which indicates a strong synergism. The results clearly demonstrated that the applied strategy for experimental design and algorithms for calculation of isobolograms under particularly chosen combination ratios was robust and very reliable. The synergistic and additive effects were confirmed by the CFU assay (Figure 3B). The effect of the combination 1:4 [ERF:MCRM] in HuT-78 cells led to complete inhibition of cell clonogenicity, and therefore, this combination was subjected to a series of tests to study its effect on the expression of the transcription factor TWIST1, PKB/Akt phosphorylation, NF-κB activity, apoptosis induction, and glutathione reduction.

Undoubtedly, an important outcome was the complete suppression of the TWIST1 expression after the application of the synergistic combination [20 μM ERF:80 μM MCRM] on HuT-78 cells (Figure 4A) compared to the single application of erufosine that, although at a concentration of 50 μM, suppressed the TWIST1 expression by only 60% (Figure 4B). The achieved effect makes it feasible to reduce the effective dose of ERF and thus minimize the occurrence of adverse effects. The same effect was observed with the MJ cell line, except that ERF alone inhibited TWIST1 by 80% at a twofold lower concentration than in HuT-78 cells (25 µM vs. 50 µM), which may explain why the effect of the combination [20 μM ERF:80 μM MCRM] is additive and not synergistic (as in HuT-78 cells). Regarding PKB/Akt dephosphorylation, the combination inhibited almost fully the protein phosphorylation at Ser473 in both cell lines, which correlates with the results from the MTT and CFU assay, suggesting that proliferation inhibition occurs via inhibition of PKB/Akt. It also confirms that the lower sensitivity of HuT-78 cells to ERF is due to the weaker inhibition of this protein than in MJ cells. A single administration of ERF (20 µM) or MCRM (80 µM) achieved this effect to a greater extent in MJ cells than in HuT-78. In MJ cells, dephosphorylation at Thr308 was more pronounced than at Ser473, which is in line with previously published data [36]. In HuT-78, there was no significant difference in the levels of p-AktThr308 between the treated samples, whereas the levels of p-AktSer473 decreased significantly and to the same extent after exposure to MCRM or the combination. The activity of NF-κB p65 was also inhibited by up to 60% in both cell lines after administration of the combination (Figure 4C). The differences between the effects of the combination and the self-administered drugs were significant. The lack of effect of MCRM on NF-κB expression can be explained by the slow, gradual release of curcumin from the particular micelles during the incubation period—35% (28 µM) of the whole loaded dose, which is 80 µM. The released amount of CRM is obviously not sufficient to achieve the desired effect for this incubation period (24 h). In contrast, according to published data, 80 µM of pure CRM or CRM loaded in nanoparticles with rapid release significantly inhibits the activity of NF-κB [41,76,82,83]. In this regard, we could assume that not the gradual administration of low CRM doses over time but the fast release of higher doses in the first hours after treatment is crucial for the NF-κB inhibition. However, in combination with ERF, the low concentrations of CRM released from the micelles were sufficient to potentiate the effect of the inhibitory effect of the alkylphosphocholine on NF-κB, suggesting the unlocking of other mechanisms that deserve further investigation in a separate study. In the MJ cell line, the effect of ERF was the same as in the combination, which correlates with the determined additive effect.

The inhibition of NF-κB and PKB/Akt logically led to the induction of apoptosis in the treated cell lines. As visible in Figure 5, the combination 1:4 [ERF:MCRM] activated caspase-3, increased the fraction of mono- and oligonucleosomes in the cytosol of HuT-78 and MJ and led to nuclear fragmentation in both cell lines, indicating apoptosis (Figure 5C). Activation of caspase-3 and elevation of the reduced glutathione levels were more pronounced in MJ cells after treatment with the combination (Figure 5A,B). One possible explanation for this difference could be an earlier induction of apoptosis in HuT-78 cells, which could be proven in detail in a further study. As far as excess GSH in malignant cells correlates with increased metastasis and tumor progression [84,85], it could be speculated that the elevated GSH levels in the MJ cell line in our study could be one of the reasons why the best effect of the combination 1:4 [ERF:MCRM] on MJ cells is not synergistic but additive only.

Last but not least, the synergistic combination fully inhibited the formation of MRSA biofilm, which can bring additional benefits for patients with concomitant staphylococcal infections as this will further help reduce inflammation and limit the appearance of infected skin lesions. ERF alone is also a potent inhibitor of the MRSA biofilm, as published before [29], and the combination with MCRM did not reduce this effect.

## 5. Conclusions

The anti-lymphoma effect of ERF in CTCL cell lines was dependent on the TWIST1 expression. The combination between ERF and MCRM in a ratio of 1:4 achieves a synergistic or additive effect depending on the cell line, which was due to inhibition of TWIST1, dephosphorylation of PKB/AKT at Ser^473^ and/or Thr^308^, and deactivation of the p65 subunit of NF-κB. Taken together, our experimental findings indicate that it is feasible to develop and investigate new micellar CRM formulations in rational combinations with the alkylphosphocholine ERF for future local and/or intravenous CTCL treatment perspectives. The low toxic profile of the two substances allows their combination to be used in a broader pharmacological regimen, with the combined antineoplastic, anti-inflammatory, and antibacterial activity assisting in overcoming primary and secondary drug resistance of CTCL-derived cells.

## Figures and Tables

**Table 1:** Extracellular concentration of MCRM in comparison to pure curcumin after treatment of HuT-78 cells.

Drug	Incubation Time [h]	Absorption (AU) +/− SD	A_sample_ − A_blank_ (AU)	Quantity of the Substance (mmol)
Control	0	0.05748 +/− 0.01	-	-
CRM		0.47531 +/− 0.01	0.41783	8.0290 × 10^−5^
MCRM		0.25410 +/− 0.01	0.19662	3.3212 × 10^−5^
Control	1	0.12833 +/− 0.02	-	-
CRM		0.38940 +/− 0.02	0.26107	4.4100 × 10^−5^
MCRM		0.27864 +/− 0.02	0.15033	2.5394 × 10^−5^
Control	2	0.10574 +/− 0.02	-	-
CRM		0.37878 +/− 0.02	0.27304	4.612 × 10^−5^
MCRM		0.23807 +/− 0.02	0.13233	2.2354 × 10^−5^
Control	3	0.15630 +/− 0.01	-	-
CRM		0.43492 +/− 0.01	0.27862	4.7064 × 10^−5^
MCRM		0.23947 +/− 0.01	0.08317	1.4050 × 10^−5^

**Legend:** AU—absorption units; CRM—pure curcumin dissolved in ethanol; MCRM—micellar curcumin.

## Data Availability

All raw data from the experiments are available from the authors.
